# Health-Care Professionals' Assessments of, and Recommendations for, Sexual-Health Education and Service Provision for Young People in Tehran

**DOI:** 10.3389/fpubh.2021.634795

**Published:** 2021-08-24

**Authors:** Narges Sheikhansari, Charles Abraham, Sarah Denford

**Affiliations:** ^1^Medical School, University of Exeter, Exeter, United Kingdom; ^2^Melbourne Centre for Behavior Change, Melbourne School of Psychological Sciences, Faculty of Medicine, Dentistry and Health Sciences, University of Melbourne, Melbourne, VIC, Australia; ^3^Children's Health and Exercise Research Centre (CHERC), University of Exeter, Exeter, United Kingdom

**Keywords:** sexual health, healthcare professional, Tehran (Iran), young adult, sexual health education

## Abstract

**Background:** Only limited Sexual Health and Relationships Education (SHRE) is provided in Iranian schools and universities while research has highlighted demand and need for improved SHRE among young adults. We explored health-care professionals' (HCPs) assessments of, and recommendations for, SHRE and service provision for young people in Tehran.

**Design and Methods:** Semi-structured interviews were conducted with a sample of 17 HCPs based in Tehran and verbatim transcripts were analyzed using thematic analysis.

**Results:** Participants confirmed the need for improved SHRE and service provision for young adults. HCPs described how a lack of reliable educational resources for young adults, taboo and cultural barriers, and a lack of trust and confidentiality prevented young people from accessing information and services. They unanimously supported education and services to be augmented, and provided recommendations on how this could be achieved.

**Conclusions:** A number of positive suggestions for the improvement of SHRE and Iranian sexual health services in Iran were identified.

## Introduction

Health care professionals (HCPs) in Iran, as in other countries, face cultural barriers to implementing evidence-based, sexual-health education and training (1). Optimal sexual health and relationship education (SHRE) for young people could challenge normative and even legal constraints in Iran. Yet STIs and abortion rates indicate that better education is needed. Available data suggests that more than 66,000 people are HIV positive in Iran, of whom, approximately 30% were infected through unprotected sexual intercourse (2, 3) and that this transmission route explains increasing numbers of cases (2, 4, 5). Moreover, illegal abortions are increasing and have been estimated to exceed more than 1,000 cases per day (6).

Tehran is the most populous city in Iran with a population of 8.5 million (in a country of 81 m) (7), including ~1.04 m people aged 18–25 (8). Young people in Iran complete schooling at 18, the same age at which they can legally marry. Tehran has a limited number of “Centers for Behavioral Diseases” that provide confidential, free testing for sexually transmitted infections (STIs) (4) but these facilities are not publicly advertised and are primarily used by “high risk” groups including drug users and sex workers (9). These clinics do not offer preventive or advice services for young people who contemplate romantic and sexual relationships before the age of 18 years [unlike e.g., (10)].

SHRE has never been included in Iranian school curricula but many Iranian universities, including those in Tehran, offer a single module (“Science of Family and Population”) which is a 20-h course taught by a lecturer specializing in religious and spiritual studies (11). The module is aimed at heterosexuals and does not include topics highlighted as best practice by The United Nations Educational, Scientific and Cultural Organization (UNESCO), such as sexual consent, prevention of sexually transmitted infections, use of available contraception options and safer sexual practices across sexual orientations (12, 13). In addition, pre-marriage classes are compulsory before marriage, but deliver similarly limited content. Researcher-lead sexual health education modules have also been reported for specific sub-groups (14), including married, Muslim women within particular healthcare settings (15). Overall, however, there is limited SHRE provision for young people in Tehran, and in Iran generally.

According to the World Health Organization (WHO), SHRE should promote “a state of physical, mental and social well-being in relation to sexuality … requiring a positive and respectful approach to sexuality and sexual relationships, as well as the possibility of having pleasurable and safe sexual experiences, free of coercion, discrimination and violence” (16). SHRE is effective. A review of reviews incorporating 37 systematic reviews (and 224 primary trials) indicated that comprehensive school-based SHRE can increase knowledge, change attitudes, and reduce risky sexual behavior (17). Denford and colleagues provide useful evidence-based recommendations for implementing optimal SHRE. Additionally, a United Nations Educational, Scientific and Cultural Organization (12) review of 85 SHRE interventions, for young people (aged 15–24), delivered in schools, community centers, and health clinics in the United States of America and developing countries, concluded that such interventions were effective and that there was no evidence showing that SHRE results in earlier or more frequent sexual encounters.

The demand for comprehensive SHRE in Iran has been documented. Shahhosseini and Hamzegardeshi (18) conducted interviews with 77 young Iranian women, aged 11–19 and concluded that there was a strong demand for SHRE and that, in its absence from school curricula, young adults have turned to unreliable internet sources (e.g., Instagram and Telegram) (14). Mosavi et al. (19) came to similar conclusions, based on semi-structured interviews with adolescent girls and their mothers. They highlighted a lack of knowledge regarding sexual health, use of unreliable and inaccurate information through the Internet, and increased risky, sexual behavior patterns among adolescents. This corresponds to our findings from interviews with young Tehranians, which showed that young people used informal sources of sexual health information, including social media and friends and that they did not trust medical consultations to be confidential. They unanimously expressed their dissatisfaction with the current health services, including preventative and educational support (20).

Despite public health need and clear demand from young people there are socio-cultural barriers to the delivery of comprehensive SHRE in Iran. Latifnejad Roudsari et al. (21) reported a qualitative study of interviews and focus group discussions with 57 students and 10 of their mothers in two large Iranian cities (Mashhad and Ahvaz). They found that social taboo, reluctance to discuss sexual matters and concerns about negative consequences were barriers to using sexual health services. This was supported in a study by Mosavi et al. (19), in which 247, 14–19 year old girls and 26 of their mothers were interviewed along with 45 key informants in four Iranian cities (Tehran, Mashhad, Shahroud, and Qom). In accordance with other literature, social and cultural challenges were the most quoted barrier to SHRE development. These participants also highlighted the Iranian legal and political structure, which only recognizes sexual health as an issue for legally married people. These barriers primarily, although not exclusively, affect women (22). Mirzaii Najmabadi et al. (23) studied a sample of 34 men, aging from 22 to 66 years, both married and unmarried. They found socio-cultural barriers, particularly the stigmatization of sexual activity, prohibits open discussion and discourages help-seeking information provision and sexual-health care.

So, given the potential effectiveness of SHRE and the lack of negative consequences observed across studies (17), the public health need, the demand especially from young people and the socio-cultural and legal barriers, what can be done to improve SHRE for young Iranians? We addressed this question through discussions with HCPs and policy makers in Tehran. Their views are important because involvement of key stakeholders and co-design of education and services is likely to optimize effectiveness (24, 25).

### The Present Study

We investigated the views of Tehran-based HCPs regarding sexual-health education and services for 18-25 year-old (young) Tehranians, We also explored the potential utility of new workshops to augment current provision. Discussion focused on five topics (full topic guide is available as a part of the Supplementary Material).

1. Young Tehranians' sexual health knowledge and behavior patterns,
2. Young Tehranians' sexual health and educational needs,
3. Current provision of sexual health and relationship education for young Tehranians,
4. Availability and accessibility of sexual health services for young Tehranians,
5. Acceptability, feasibility and the optimal delivery of new workshops to augment current provision.

## Methods

Semi-structured interviews were conducted with male and female HCPs and policy makers in locations convenient to them within Greater Tehran. The study was approved by ethics committees of both The University of Exeter Medical School and Iran University of Medical Sciences and conducted in accordance with the Standards for Reporting Qualitative Research (26).

### Sampling and Data Collection

Purposive sampling was used to recruit a range of HCPs working in public and private health sectors in Tehran. Some had direct contact with patients/clients while others did not. All were experts in sexual health and/or were responsible for designing and delivering sexual health services, interventions and/or policies.

Inclusion criteria were being Iranian, based in Tehran, speaking Persian as a first language and having direct or indirect relationship with sexual health initiatives delivered in Tehran, including policy making and client advisory services. The sample was recruited using a snowballing procedure in which participants were asked to provide names of other HCPs meeting our criteria. These were telephoned and, if interested, provided with information about the study and invited to participate. Those who consented were asked for a convenient interview time and location. Participants were informed that they could withdraw at any time without providing a reason. Verbal consent (rather than written consent) was obtained from every interviewee to protect anonymity. All participants consented to interviews being recorded and for quotations to be reported anonymously.

### Participants

Twenty-nine HCPs were contacted. Twelve declined or were unavailable during the data collection period. Seventeen Iranian, Persian-speaking HCPs working in Greater Tehran were interviewed (5 men and 12 women). These included HCPs with and without medical qualifications and those in clinical contact with patients and clients (*N* = 7) and social workers and community health consultants (10) of whom seven were policy makers or had a high level of influence on policy development and implementation in relation to sexual health.

### Interviews

A topic guide was developed to plan semi-structured interviews (Supplementary Material 1). The guide included questions on HCPs perception of (1) young Tehranians' sexual health knowledge (2) young Tehranians' confidence in preventing STIs and protecting themselves in sexual relationships (3) content and quality of existing sexual health edu-cation (4) sources of sexual health information for young Tehranians (5) recommendations for improved provision of sexual health education in Tehran, (6) current sexual health services and (7) accessibility of sexual healthcare and contraceptives. The topic guide was revised after pilot testing with 5 HCPs. Interviews were completed in Persian language and recorded interviews were transcribed verbatim, anonymized and translated into English.

### Data Analysis

NVivo was used to store transcripts and allocate excerpts to categories. Thematic analysis was undertaken using Braun and Clark's (27) guidelines. This involved 5 stages of analysis of anonymized interview transcripts, including (1) becoming familiar with the data (reading, re-reading and note taking) and (2) generating preliminary category definitions (outlining definitions and identifying content examples and duplicate categories). Step 3 consisted of a more systematic search for themes and overlapping/corresponding thematic definitions (e.g., checking if emerging definitions were applicable across interviews or were too general/specific). In step 4 the developing thematic/sub-thematic definitions were revised (e.g., ensuring that defined categories were distinct and represented multiple examples across interviews). In step 5, we finalized thematic definitions and the conceptual tree in which themes were situated. Steps 3–5 involved multiple meetings between three researchers to critically discuss theme and sub-theme definitions and the allocation of quotes and the relationships between themes. Finally, transcripts were re-read and reviewed to ensure that the selection of quotes was appropriate and comprehensive. The first author made reflective notes during data collection and referred to these during analyses to ensure that participants' views were captured accurately.

## Results

Analyses extracted 231 quotations from 17 transcripts, representing ~80% of transcript text. Thematic analyses generated 11 themes, incorporating 28 sub-themes. Theme names and the number of quotes representing each theme are presented in Table 1. All extracted quotations are presented, by theme and sub-theme, in the Supplementary Material. Below we explain the meaning of each theme and sub-theme. Representative quotes are presented in Table 2.

**Table 1:** Main themes.

**Theme name**	**Number of quotes**
1. Sexual Health Needs	9
2. Cultural and Social Barriers to Optimal Sexual Health Practices	31
3. Current Sexual-Health Educational Provision	8
4. Limitations of Current Sexual-Health Educational Provision	49
5. Informal Sexual-Health Education and its Limitations	27
6. Sexual Health Services for Young People	8
7. Barriers to Seeking Sexual Healthcare	26
8. Recommendations for Improved Sexual-Health Education and Services in Tehran	16
9. Support for a New SHRE Workshop	12
10. Content Suggestions for a New SHRE Workshop	33
11. Workshop Delivery Suggestions	12

**Table 2:** Illustrative quotes by Theme and Sub-theme.

**Theme 1: Sexual Health Needs**
*Sub-theme 1a: Increasing STIs Prevalence* Nowadays HPV has become very common, so are herpes and vaginal infections, because multi partnership is more common. *Sub-theme 1b: Clients' Concerns About Unintended Pregnancy* If there's any concern it is about pregnancy, not STIs. They are concerned about getting pregnant because they have no idea what they should do. It's a big problem, they don't know how to solve.
**Theme 2: Cultural and Social Barriers to Optimal Sexual Health Practice**
*Sub-theme 2a: The Iranian Legal Context* There's no legal support [for young people having sex out of marriage]. Pregnancy becomes obvious … so getting an abortion will become the person's only option. [This] leads to unsafe abortion that unfortunately happens a lot in Iran. Abortion is not legal in Iran unless mother's health is at risk or the fetus has a serious problem. *Sub-theme 2b: Social Norms and Taboo* It hasn't become normal in our culture for people to seek healthcare for their sexual issues. Our society hasn't reached that level of insight that looks at this [sexual health] as a normal thing in daily life that needs to be taken care of…. It is considered a taboo even by educated people. *Sub-theme 2c: Gender Inequalities* Women don't usually choose not to use condoms. They might try to teach their partners about some behaviors and see what is comfortable for them however it doesn't help much. Men feel they always know it all. *Sub-theme 2d: Pre 2008/2013 Policy and Services* All services that sexual health centers provide used to be free. But following policies aimed at increasing the population we don't offer contraceptives and condoms for free anymore. HIV used to be taboo, you couldn't talk about it comfortably, it was the same for STIs. In this golden era [2006-7] this taboo was broken [so that] they were discussing teams that would go to different areas of the city and provide free HIV tests for young adults. Now it's all gone.
**Theme 3: Current Sexual-Health Educational Provision**
We hold pre-marriage classes here. 2 hours of these classes are dedicated to sexual and pregnancy health. Around 90 minutes for ethics and religious rules, 45 minutes of legal rights and 90 minutes is dedicated to psychology. We recruit high risk and at risk young adults. [The project] is developed by UNICEF's “All In Project” [and] located in “a deprived area. We were the first such project across the Middle East. Its name is “Youth Health Center”, 50% of its funding comes from UNICEF and the other 50% from the national budget. This program teaches young adults about HIV.
**Theme 4: Limitations of Current Sexual Health-Educational Provision**
*Sub-theme 4a: Lack of Sexual Health Knowledge* Most young adults who come to my clinic don't know even the most basic information…. one of the common questions that I get is what contraception method should they use, and sometimes they ask how should they have sex, what position is better… They know almost nothing about STIs. Most of our referrals come here [Center for Behavioral Diseases] for STIs testing, HIV specifically. Yet, they have no information whatsoever, regarding STIs or pregnancy or anything else related to their sexual and relationship health. *Sub-theme 4b: Lack of Self and Relationship Management Skills* I think no one has the ability to manage their sexual relationships as they never been taught or encouraged to do so. *Sub-theme 4c: Pre-Marriage Provision* I never see a difference between my married and unmarried patients in terms of sexual health knowledge or safer sexual behavior. That may mean that these classes are probably nonsense. I haven't gone to check what they teach. Such classes should take place much earlier so people [do not] do things without knowing what's the right thing to do. *Sub-theme 4d: Organizational and Cultural Constraints on Improved SHRE* We don't get enough budget to set up as many centers with enough trainers and experts. Our education system doesn't let us go to schools to educate students about this stuff, the reason they give us is that the parents might complain about why did you tell this stuff to our children? Most of the decisions regarding this age group are being made by people who are from other generations and might not understand their issues. W we need to communicate with them [young adults] to understand their real problems. *Sub-theme 4e: Lack of Formal Evaluation of Programs and Content* We have opened centers for behavioral diseases but we have never assessed how many people know of these centers or use them.
**Theme 5: Informal Sexual Health Education and Its Limitations**
*Sub-theme 5a: Friends* Young adults know some stuff but not much, and they have learned it from their friends. They only use withdrawal. They only do it because their friends do it. *Sub-theme 5b: Internet and social media* All the information is on social media. They will learn where they can find the most reliable information. Society is helping itself with no official support. But the internet is like an ocean, anything could be found in it and many sources of information could be unreliable or misleading.
**Theme 6: Sexual Health Services for Young People**
*Sub-theme 6a: Services in Center for Behavioral Diseases* When people come here to get tested, they first go through a counseling session and then we test them for HIV based on the risky behaviors they've told us about. If the result of the rapid test is positive they are sent for further clinical tests…. Other STIs are not screened in this center. People need to go to a specialist if they have other STIs symptoms. *Sub-theme 6b: Funding Limitations* The health budget is mostly focused on high risk groups. For the general population there are not much done in terms of sexual health. Unfortunately, at the moment all of our sexual health services, which are limited compared to the other countries, are only tailored for the high-risk groups.
**Theme 7: Barriers to Seeking Sexual Healthcare**
*Sub-theme 7a: Lack of Publicity for Government Funded Sexual Healthcare Facilities* Regarding sexual healthcare facilities, there might be some centers but I don't know about them. *Sub-theme 7b: *Costs** Those who can afford it, refer to private clinics and doctors, and receive all care needed, at times even illegally, like abortion. Those who can't, the absolute majority, suffer in silence. *Sub-theme 7c: Inequalities in Sexual Health and Care Seeking* We are in an affluent area of Tehran so normally I would expect people who refer to us to have a better level of knowledge in sexual health. They do have more knowledge in comparison to those who live in deprived areas. *Sub-theme 7d: Distrust in Available Services* Also they don't trust these services [e.g., centers for behavioral diseases], they don't trust them to be confidential and non-judgmental. It means people find internet and their friends trusted sources instead of us. That's sad, but once we have lost this trust, we can't gain it back so easily.
**Theme 8: Recommendations for Improved Sexual Health Education and Services in Tehran**
*Sub-theme 8a: Creation of Official Sources of Information* Policy makers… don't want face communication about sex. Fine, tell our children what online source is reliable and educative and they will find [out for themselves] *Sub-theme 8b: Official School/University Based SHRE* We need… rigorous sexual health education right after elementary school. We need to teach a comprehensive course and let them ask all their questions. But I'm not the one who sets policies, unfortunately.
**Theme 9: Support for a New SHRE Educational Workshop**
Everyone would love to attend such a workshop. There are no alternatives. This is like a fresh air, a new idea. Something they have really wanted for so long.
**Theme 10: Content Suggestions for a New Workshop**
*Sub-theme 10a: Anatomy of Sexual Organs* The first thing that needs to be taught is the anatomy of sexual organs. I would explain it in a way that is simple and easy to understand. They should learn about what happens during sex and how pregnancy occurs. *Sub-theme 10b: Pregnancy Prevention, STIs Protection and Condom Use* How conception happens, how to aid or avoid it, how to keep healthy and avoid STIs. Who to go to to fix things [if they contract an STI]. What… protection methods are available and why is it so important to buy, carry and use condoms. *Sub-theme 10c: Provision of Contact Details for Available Sexual Healthcare* Include addresses and contact details of [sexual health] centers. *Sub-theme 10d: Self and Relationship Management Skills* Management of sexual relationships… is really important
**Theme 11: Workshop Delivery Suggestions**
*Sub-theme 11a: Mixed or Single Gender Classes* These are sensitive subjects and not everyone is comfortable with them. You will need to ask your audience whether they want to be in mixed or single gender settings. *Sub-theme 11b: *Group Discussions and Q&A** Let them ask any questions they have and let them be as open as they wish to be. It's their first chance in their lifetime.

Demographic data including age, gender, socioeconomic status, and self-expressed religious beliefs were recorded, but we did not find patterns in HCPs responses that were attributable to these characteristics and, therefore, these are not included the results section.

### Sexual Health Needs

Participants expressed concern about increasing numbers of STIs identified amongst young people in Tehran, especially HPV and Genital Herpes and inferred that these represented new patterns of more risky sexual behavior (sub-theme 1a). A few participants also highlighted concerns about unintended pregnancy, emphasizing that these concerns were often more important to young people than those relating to STIs because of the social visibility of pregnancy and the socio-cultural context (1b).

### Cultural and Social Barriers to Optimal Sexual Health Practice

Participants highlighted a range of socio-cultural barriers, including the lack of legal and service support for individual sexual choices (2a). Participants noted that the illegality of sexual relations outside marriage and of abortion, created fear and repressed open discussion of safer sexual practices and service use among unmarried people (2b). This prevents the normalization of sexual healthcare for unmarried people.

Participants also discussed gender power inequalities, highlighting disempowerment of women in heterosexual relationships and the implications for contraceptive use and STI-preventive practices. The challenge here is to provide support for young women to take responsibility and skillfully manage their sexual health (2c).

Some participants highlighted 2013 changes in national policy and their healthcare implications. They explained that a successful national family planning program had been in place between 1998–2006, which involved free and unlimited access to condoms and contraception methods including tubectomy and vasectomy. This was largely withdrawn in 2013 as national policies focused on increasing Iran's population (2d).

### Current Sexual-Health Educational Provision

Participants commented on current provision, including HIV-awareness courses delivered in high schools and STI-awareness courses delivered in universities. Participants were, unsurprisingly better informed about educational initiatives run by their own organizations but these various programs are not officially evaluated and the number of users/recipients is unknown. Overall, HPCs painted a picture of a patchy educational service with some centers of excellence but poor provision for the general population of young people.

### Limitations of Current Sexual-Health Educational Provision

Almost all interviewees agreed that sexual-health knowledge was poor among their young patients/clients. A concerning lack of understanding of sex, contraception, and STIs was reported. Interviewees attributed this to a lack of reliable information sources, including official sexual-health education (4a). This was combined with poor self and relationship management skills among young Tehranians due to limited interpersonal communication skills-training during their education (4b).

Participants were critical of existing pre-marriage classes in terms of SHRE delivery, including content range, delivery methods, timing of the intervention, and lack of evaluation (4c) and supported more comprehensive, more accessible SHRE including workshops. However, they also recognized that cultural constraints render such developments problematic, including parental objections, and policy-makers lack of understanding of the sexual-health needs of young people (4d). Participants also highlighted a gap between what policy-makers and legislators deem necessary and the education that front-line HCPs view as essential to protect health and promote wellbeing and noted that current provision is not usually evaluated in terms of reach, use, or impact (4e).

### Informal Sexual Health Education and Its Limitations

Two clear sources of sexual-health information for young Tehranians were identified; friends (5a) and the internet (5b). Both were considered to be popular but were acknowledged as sources of unreliable information that could engender potentially wide-spread misinformation and sub-optimal sexual practices (such as using withdrawal as a contraception method).

### Sexual Health Services for Young People

Sexual healthcare services offered by “Centers for Behavioral Diseases” (CBDs) (6a) were discussed by interviewees. In Iran, general health centers resemble a GP office or clinics for family medicine where general health and well-being concerns are addressed, while CBDs provide specialized STI services. Participants noted the lack of preventive services and one-stop-shop services (e.g., most CBDs only focus on HIV). The lack of preventive services was lamented and generally attributed to limited funding (6b).

### Barriers to Seeking Sexual Healthcare

Almost all participants noted the lack of publicity for government-funded sexual healthcare, including CBDs (7a) and that cost was a major barrier (7b), especially since healthcare is mostly privatized in Iran, highlighting socioeconomic inequalities in health care access (7c), especially for young Tehranians many of whom do not have secure incomes.

Participants also noted that young people distrust official sexual health services (7d) fearing that confidentiality will not be respected and that doctors may be judgmental.

### Recommendations for Improved Sexual Health Education and Services in Tehran

There was consensus for two important changes needed to improve SHRE education and services for young people. First, participants recommended that, in the absence of comprehensive SHRE, officials recognize young people's use of the internet as an educational resource and offer endorsement and recommendation of reliable online educational resources (8a). Second, participants recommended development of comprehensive SHRE in secondary schools and university across Iran (8b). Although, there was pessimism about this being accepted as future policy.

### Support for a New SHRE Workshop

Participants believed that a new SHRE workshop or day-course would be beneficial. All HCPs unanimously supported such new SHRE workshop and 12 of them provided longer quotes, which are included in the Supplementary Material.

### Content Suggestions for a New SHRE Workshop

Anatomy of sexual organs was considered essential by many participants as this subject is not fully covered in human biology courses in schools (10a). STIs prevention, use of condoms and other contraceptives for pregnancy prevention was suggested by all participants (10b). In addition, wider publicity for existing services (10c), and relationship management skills (10d) were highlighted as important content.

### Workshop Delivery Suggestions

Participants were divided on the merits of same vs. mixed gender classes (11a). Some believed mixed classes would facilitate breaking taboos while others felt that same-gender classes would provide a more relaxed learning environment, especially for young women. Participants supported group discussions and Q&A opportunities (11b).

## Discussion

To our knowledge, this is the first qualitative investigation of HCPs' views of sexual health education, training, and service provision for young Tehranians. Together with previous studies of young Iranians' views of SHRE and sexual-health services in Iran, our findings highlight a serious public health need and a potentially culturally acceptable way forward. In particular, (i) official endorsement of selected online resources and (ii) provision of new culturally-tailored, government-endorsed workshops for young Iranians focusing on knowledge transfer, STIs and pregnancy prevention and relationship management skills are recommended.

Confirming previous research, HCPs suggested that a lack of SHRE in Iran contributes to increasing STIs and illegal abortions (28). Interviewees were critical of existing initiatives, which predominantly focus on HIV awareness, and do not cover core content such as contraception and relationship management skills. HCPs clearly identified a lack of governmental support and funding as the key challenge to developing more comprehensive SHRE, similar to previous conclusions made by Yazdanpanah et al. (29) and Mehrolhassani et al. (30).

Despite the availability of a small number of government-funded sexual healthcare clinics, HCPs believed that (i) most young Tehranians are unaware of these facilities and, perhaps more importantly, (ii) these services do not provide preventative content. Interviewees also noted the high cost of medical consultations which exaggerates existing socioeconomic (SES) inequalities. This is particularly problematic as the majority of the population cannot afford private healthcare. Furthermore, HCPs were aware of a lack of trust among many young people; suggesting that many fear breaches of confidentiality; which would ultimately damage their social image, even in conversations with doctors. HCPs highlighted the need for improved, well-publicized, accessible, confidential and affordable sexual healthcare in combination with a comprehensive SHRE program.

HCPs made a series of recommendations for future services. In particular, SHRE that clearly explains the physiology and function of sexual organs and the mechanism underlying STIs transmission and pregnancy. In addition, they emphasized the importance of training young people in skills such as relationship management, condom-acquisition, condom-use negotiation, and consistent condom use. Participants also recommended training materials specifically addressing support for women to manage sexual relationships more confidently and to control their sexual health including instructions on how to best negotiate condom use, confirming findings of Mirzaii Najmabadi et al. (23).

HCPs noted that young people depend on unreliable educational sources, including their friends and social media, and are consequently largely uninformed and unskilled, confirming previous findings (20, 28). Participants recommended the creation of culturally tailored, government-endorsed reliable online training materials in Persian language as an initial step to bridging the identified SHRE gap. Creation of culturally-appropriate SHRE materials has been evaluated in other countries, such as Turkey, and found to increase sexual health knowledge amongst young adults, while also reducing myths and sexist beliefs and behaviors (31). It is reasonable to expect that similar positive outcomes would be see in Iran.

Interviewees noted that existing educational initiatives focus predominantly on HIV awareness, and do not include content on contraception or relationship management skills. They highlighted organizational constraints including limited funding for sexual health educational programs and lack of cooperation between various ministries and organizations responsible for such programs. The lack of evaluation of existing programs was also discussed. This casts doubt on the efficacy and reach. There was a sense of pessimism among our HCPs because they, themselves, cannot transform the sexual health services. Improvements will require operational and political decisions by national level managers and policymakers. Nonetheless, an understanding of the views of HPCs provides guidance on priorities for improvement to SHRE and sexual health services in Iran.

Moreover, HCPs considered the quality and content of existing programs, including courses tailored to marrying couples, to be ineffective and provided recommendations for how the current provision should be improved or augmented. This indicated their perceived need and demand for provision of comprehensive SHRE [cf. (29, 30)].

In spite of limited government-funded sexual healthcare clinics, HCPs believed that young Tehranians are unaware of these facilities and viewed visits to private doctors as limited by cost. They therefore recommended publicizing such services to all social groups including young adults. Interviewees mentioned lack of trust in available services and believed that young Tehranians doubt their confidentiality and fear the potential damage to their social standing, even in conversations with doctors.

In general, the results suggest a need for improved, well-publicized, accessible, confidential, and fairly priced sexual healthcare. Additionally, co-creation and testing of comprehensive SHRE modules was strongly supported.

We recruited a unique mix of private and public sector HCPs from different managerial and client-serving backgrounds. Their opinions help to draw a robust evidence-based picture of sexual health knowledge and practices among young Iranians. Our findings provide a novel needs assessment with helpful recommendations to improve sexual health services and education. Nevertheless, there are limitations to this research. A small sample was used and the views of important stakeholders (e.g., clergies and religious leaders) were absent from this study. Nonetheless, our findings support those of Akbari et al. (32), who included Iranian religious leaders in their study and recruited a larger number of participants, indicating that there is a consensus across stake holders about needed SHRE and service improvement. Thus, our study presents useful recommendations for improved SHRE and sexual health services for young Iranian adults, adding a new voice to previous studies focusing on the views of young people.

In conclusion, HCPs judged young Tehranians' sexual health knowledge to be poor and believed that existing government-funded sexual healthcare is lacking and inaccessible. They strongly suggested that available sexual health provision should be augmented and recommended development of non-judgmental, accessible, confidential and fairly-priced sexual healthcare, availability of publicized sexual health clinics, as well as provision of culturally-tailored, government-endorsed reliable online training materials in Persian. These recommendations complement and correspond to suggestions made by young Iranians, across studies. Collectively this body of research recommends action to develop new culturally acceptable SHRE for young Iranians.

## Data Availability Statement

The original contributions presented in the study are included in the article/Supplementary Material, further inquiries can be directed to the corresponding author/s.

## Ethics Statement

The studies involving human participants were reviewed and approved by The University of Exeter Medical School and Iran University of Medical Sciences. Written informed consent for participation was not required for this study in accordance with the national legislation and the institutional requirements.

## Author Contributions

NS and CA collaboratively designed the study. NS collected and analyzed the data. CA and SD advised on data analysis, participated in drafting the article, and critically revised the final version. All authors have read and approved the final version of the article for publication.

## Conflict of Interest

The authors declare that the research was conducted in the absence of any commercial or financial relationships that could be construed as a potential conflict of interest.

## Publisher's Note

All claims expressed in this article are solely those of the authors and do not necessarily represent those of their affiliated organizations, or those of the publisher, the editors and the reviewers. Any product that may be evaluated in this article, or claim that may be made by its manufacturer, is not guaranteed or endorsed by the publisher.
