# Supplementary material for: Health-Care Professionals' Assessments of, and Recommendations for, Sexual-Health Education and Service Provision for Young People in Tehran
Source: Front Public Health. 2021 Aug 24;9:634795. doi: 10.3389/fpubh.2021.634795 (PMC8421759; doi:10.3389/fpubh.2021.634795)
Supplement: Supplementary file 1 [file Table_1.DOCX]

Supplementary Documents : Health-care professionals’ assessments of, and recommendations for, sexual-health education and service provision for young people in Tehran

Contents Page

Supplementary Document 1: Interview Protocol 2-4

Supplementary Document 2: 11 Main Themes 5

Supplementary Document 3: Thematic Map 6-9

Supplementary Document 4: Quotes grouped by themes and sub-themes 10-47

Supplementary Document 1: Interview Protocol

Interview Topic Guide for:

Health-care professionals’ assessments of, and recommendations for, sexual-health education and service provision for young people in Tehran

1. I am going to ask you questions about sexual health education and training for young adults in Tehran. Do you also understand that I will audio record the interview and that you can refuse to answer any question and end the interview at any time? Do you wish to continue?
2. What is your area of expertise and how does it relate to sexual health education and sexual health care for young adults in Tehran?
3. How well educated do you think young adults in Tehran are in relation to sexual health (e.g., in relation to prevention of unwanted pregnancy and sexually transmitted infections)?
4. Are you aware of any common misconceptions or gaps in sexual health knowledge among young adults in Tehran?

a. Can you describe these?

1. How adequate/comprehensive do you think sexual health services are for young adults in Tehran?
2. Are you aware of any health problems arising from inadequacies in sexual health knowledge or services among young adults in Tehran?
3. How concerned do you think young adults are in Tehran are about unwanted pregnancy and/or sexually transmitted infections?
4. What do you think about available sexual health education for young adults in Tehran, including pre-marriage classes and university modules?
5. Do you think sexual health education in Tehran needs to be extended or changed?

a. If so, how?

1. What do you think about available sexual health services for young adults in Tehran?

a. Do you think these are known of and accessible to most young adults in Tehran?

1. Do you think sexually active young adults are in Tehran are protecting themselves against sexually transmitted infections?

a. What protection do you think they use?

1. How confident and in control do you think a typical 18-25 Tehranian 18-15 year old is in managing sexual relationships?
2. Can you identify barriers to young adults seeking sexual health knowledge and/or sexual healthcare in Tehran?
3. Do you think sexual health care (including contraception pills, condoms, educational materials, visits to doctors etc.) for young adults in Tehran is inexpensive or expensive?

a. Do you think the cost is justified?

1. What are your top five recommendations for improved sexual health education and training in Tehran?
2. Do you think a drop-in morning or afternoon workshop for young adults in Tehran on protection against sexually transmitted infections and unwanted pregnancy would be a good idea?

a. If such a workshop were to be run what would you recommend as the top five content areas it should cover?

1. Would you be willing to advise further on the content of such a workshop?
2. Would you like to add anything further?
3. Is there anyone else working in this field in Tehran you would recommend we try to contact?

Supplementary Document 2: 11 Main Themes

1. Current Sexual Health Needs
2. Cultural and Social Barriers
3. Current Sexual Health Educational Provision
4. Limitations of Current Sexual Health Educational Provision
5. Informal Sexual Health Education and Their Limitations
6. Sexual Health Services for Young People
7. Barriers to Seeking Sexual Healthcare
8. Recommendations for Improved Sexual Health Education and Services in Tehran
9. Support for a New Workshop
10. Content Suggestions for a New Workshop
11. Workshop Delivery Suggestions

Supplementary Document 3: Thematic Map

Below, the main themes identified from the analyses are described and all sub-themes are listed. There were 11 themes and 28 sub-themes.

**1. Current Sexual Health Needs** (two sub-themes)

*Increasing numbers of patients in Tehran diagnosed with various STIs, especially HPV and Genital Herpes were discussed. Interviewees inferred that these represented new and riskier sexual behavior patterns. Some healthcare professionals were also concerned about unintended pregnancy worries amongst clients. Quotes were categorized into two sub-themes.*

i. Increasing STIs Prevalence

ii. Clients’ Concerns About Unintended Pregnancy

**2. Cultural and Social Barriers** (four sub-themes)

*A number of cultural and social barriers faced by young adults when accessing sexual health services were mentioned by interviewees. Participants highlighted the lack of legal support for individual sexual choices as a barrier to such services which reiterates social taboos and stigmas. Quotes were categorized into four sub-themes.*

i. The Iranian Legal Context

ii. Social Norms and Taboo

iii. Gender Inequalities

iv. Pre 2008 / 2013 Policy and Services

**3. Current Sexual Health Educational Provision**

*Provision of sexual health interventions, including HIV awareness courses delivered in high schools and STIs awareness courses delivered in universities were discussed. These various programs are not officially evaluated and the number of users/recipients is unknown.*

**4. Limitations of Current Sexual Health Educational Provision** (five sub-themes)

*Various limitations of the current sexual health provision were discussed by interviewees. These covered different areas, including organizational, political and structural limitations. Quotes were categorized into five sub-themes.*

1. Lack of Sexual Health Knowledge

ii. Lack of Self and Relationship Management Skills

iii. Pre-Marriage Provision

iv. Organizational and Cultural Constraints on Improved SHRE

v. Lack of Formal Evaluation of Programs and Content

**5. Informal Sexual Health Education and Their Limitations** (two sub-themes)

*Healthcare professionals shared their opinion regarding young adults’ sources of education. Quotes were categorized into two sub-themes.*

i. Friends

1. Internet and social media

**6. Sexual Health Services for Young People** (two sub-themes)

*Sexual healthcare services offered by “Center for Behavioral Diseases” were discussed by interviewees. Quotes were categorized into two sub-themes.*

i. Services in Center for Behavioral Diseases

ii. Funding Limitations

**7. Barriers to Seeking Sexual Healthcare** (four sub-themes)

*Socioeconomic inequalities and expensive doctor visits were mentioned as major barriers to accessing sexual healthcare. Quotes were categorized into four sub-themes.*

i. Lack of Publicity for Government Funded Sexual Healthcare Facilities

ii. Costs

iii. Inequalities in Sexual Health and Care Seeking

iv. Distrust in Available Services

**8.** **Recommendations for Improved Sexual Health Education and Services in Tehran** (two sub-themes)

*Interviewees recommended two main solutions to improve sexual health amongst young adults. Quotes were categorized into two sub-themes.*

1. Creation of Official Sources of Information

ii. Official School / University Based SHRE

**9.** **Support for a New Workshop**

*It was unanimously believed that an educational workshop/day course would be a beneficial intervention in the absence of officially provided SHRE.*

**10.** **Content Suggestions for a New Workshop** (four sub-themes)

*Interviewees commented on headlines and subjects that need to be covered in a potential educational intervention (e.g.; a day course or a workshop). Quotes were categorized into four sub-themes.*

1. Anatomy of Sexual Organs

ii. Pregnancy Prevention, STIs Protection and Condom Use

iii. Provision of Contact Details for Available Sexual Healthcare

iv. Self and Relationship Management Skills

**11. Workshop Delivery Suggestions** (two sub-themes)

*Recommendations were provided on ways to deliver a workshop/day course. Quotes were categorized into two sub-themes.*

i. Mixed or Single Gender Classes

ii. Group Discussions and Q&A

Supplementary Document 4: Quotes grouped by themes and sub-themes

Below all quotes extracted from the interviews are categorized into the identified themes and sub-themes. Overall these quotes represented 80% of the text in transcribed interviews. The number of quotes in each of the 11 themes was as follows:

Current Sexual Health Needs [9], Cultural and Social Barriers [31],Current Sexual Health Educational Provision [8], Limitations of Current Sexual Health Educational Provision [49], Informal Sexual Health Education and Their Limitations [27], Sexual Health Services for Young People [8], Barriers to Seeking Sexual Healthcare [26], Recommendations for Improved Sexual Health Education and Services in Tehran [16], Support for a New Workshop [12], Content Suggestions for a New Workshop [33], Workshop Delivery Suggestions [12].

The following annotation is used: Participant [P], Female [F], Male [M], General practitioner [GP], Obstetrician and gynecologist[OBGYN], Infectiologist [INF], Psychiatrist

[PSY], Urologist [URL], Private Practice [PP], University professor [UP], University Associate Professor [UAP], PhD [PhD], Master of public health [MPH], Masters [MS], Bachelors [BA], Medium managerial level [MML], High managerial level [HML], Moderate influence on policy making [MPI], High influence on policy making [HPI], practice located in High Income area (HI) and practice located in Middle Income (MI).

**1. Current Sexual Health Needs**

Mainly in response to questions 3, 4, 6 and 7 from the interview protocol.

3. How well educated do you think young adults in Tehran are in relation to sexual health (e.g., in relation to prevention of unwanted pregnancy and sexually transmitted infections)?

4. Are you aware of any common misconceptions or gaps in sexual health knowledge among young adults in Tehran?

a. Can you describe these?

6. Are you aware of any health problems arising from inadequacies in sexual health knowledge or services among young adults in Tehran?

7. How concerned do you think young adults are in Tehran are about unwanted pregnancy and/or sexually transmitted infections?

1i. Increased Number of STIs

If I compare the present day to the past, I would say in the past 20 years, it is so obvious that number of patients with HPV has increased significantly and HPV has spread in a large scale, another STI that has become common and widely spread is genital herpes while it all could be prevented by using condoms and right education. There’s vaccines for HPV but people and families need to get educated and encouraged to use it…. Unfortunately, now we are dealing with a wave of HIV infections from unprotected sex because there is no sexual education. (P1, F,60, OBGYN, UP, HPI, HML, MC)

10 or 15 years ago HPV wasn’t this common, it existed but not this much. We used to see patients who had herpes here and there but it was really rare. But nowadays these 2 STIs have become really common. I mean among the patients that I visit daily that could be around 12 to 17 patients a day, in a week I might see 4-8 patients with HPV/Herpes. For instance, I would definitely tell a young girl who is 18 or 19 to get vaccinated for HPV. Before I didn’t see much need for it, especially because it’s expensive, but now I tell everyone. (P2, M,57, URL, PP, MC)

I’ve seen so many cases of HPV, not so many gonorrhea cases but we have lots of cases of chlamydia. 20 years ago no one would come to visit me for such STIs. It’s crazy how many STIs cases we see nowadays. Seems like there is so much unprotected sex going on. (P4, F,60, OBGYN, UAP, PP, UM)

HPV is scarily on the rise. Some of our health centers’ staff tell us that from every 2 or 3 patients 1 is HPV positive. It is among our young adults and teenagers in particular. (P6, F,42, PhD)

Nowadays HPV has become very common, so are herpes and vaginal infections, because multi partnership is more common (P9, F,47, OBGYN, PP, U)

Herpes and HIV specifically have grown rapidly. (P13, M,42, INF, HPI, HML)

1ii. Clients’ Concerns About Unintended Pregnancy

[They worry about it] Obviously because pregnancy is something the public will see and judge. (P4, F,60, OBGYN, UAP, PP, UM)

They are most concerned about unintended pregnancy and getting STIs would be the second if I want to put them in order. They might be afraid of what happens next and fear of the probable consequences. Yes, I’m pregnant and unmarried, now what should I do? (P1, F,60, OBGYN, UP, HPI, HML, MC)

In my opinion if there’s any concern it is for pregnancy and not STIs. They are concerned about getting pregnant because they have no idea what they should do next. It’s a big problem to them that they don’t know how to solve it. (P6, F,42, PhD)

**2. Cultural and Social Barriers**

Mainly in response to questions 12 and 13 from the interview protocol.

12. How confident and in control do you think a typical 18-25 Tehranian 18-15 year old is in managing sexual relationships?

13. Can you identify barriers to young adults seeking sexual health knowledge and/or sexual healthcare in Tehran?

2i. The Irania legal context

There’s no legal support [ for young adults having sex out of marriage]. Pregnancy would become obvious gradually and should be dealt with so getting an abortion will become the person’s only option and then it leads to a likely unsafe abortion that unfortunately happens a lot in Iran. Abortion is not legal in Iran unless mother’s health is at risk or the fetus has a serious problem. (P1, F,60, OBGYN, UP, HPI, HML, MC)

Men are not like this [afraid of consequences of sex out of marriage] because society and law is on their side. (P6, F,42, PhD)

[Commenting on reasons for fear of unintended pregnancy] Maybe because of our culture which is very male oriented. Another reason could be the lack of legal support for sex out of marriage. (P2, M,57, URL, PP, MC)

The health system is a barrier in itself. You visit a health center and you’re asked whether you’re married or not. We all know sex out of marriage is illegal, if you go and lie and say yes I am married, they want your husband’s confirmation for providing you with IUD or tubectomy. If you say I’m not married, they won’t give you the service, let alone they might be judgmental or might call the police. Why would a sane person put themselves in such a position of distress and humiliation? (P3, M,55, GP/MPH, HPI, HML)

If there was any legal support maybe they [young adults] could have overcome these situations. At least when you were in a difficult or unpleasant sexual relationship, you knew you could be supported by law. But our legal system does not recognize domestic violence as a thing and premarital sex is even worse to them. So people just keep quiet and try to deal with it themselves. (P3, M,55, GP/MPH, HPI, HML)

Maybe if there was a legal support and protection this fear [of unwanted pregnancy] would decrease. (P4, F,60, OBGYN, UAP, PP, UM)

We don't have any legal support for sex out of marriage and its consequences. Do you know how many cases of civil partnership we have now? I didn’t see such a thing back then [when I first started working]. The thing that I see a lot nowadays is that couples live together without getting married, and one of their main issues is how to have a baby. I tell them to first go and see a lawyer to find out who will be the baby’s legal guardian. I ask them not to have a baby before understanding this so that they don’t put themselves and the baby under stress. (P4, F,60, OBGYN, UAP, PP, UM)

If a legal support system existed, we would have had to accept. For example, the person chooses to have sexual relationship before marriage which is punishable under the current law. So instead of legal supports there’s penalty. Therefore, in this situation you can’t expect any authority to provide education for young adults [ who are majorly unmarried]. (P5, F,45, MS, MPI, MML)

2ii. Social Norms and Taboo

It hasn’t become normal in our culture for people to seek sexual healthcare for their sexual issues. (P10, F,48, MS)

Sex and its related matters are still a taboo here. Talking about it is yet to become normal. (P12, F,44, OBGYN, PP, MC)

Our society hasn’t reached that level of insight that looks at this subject [sexual health] as a normal thing in their daily life that needs to be taken care of. The majority haven’t reached that point. It is considered a taboo even by educated people. (P12, F,44, OBGYN, PP, MC)

This topic is a taboo, and that's why they [young adults] don’t learn these stuff from parents. (P2, M,57, URL, PP, MC)

It is the society. For example, if you are HIV positive you can’t convince anyone that you got it from a dentist visit or a tattoo artist. (P2, M,57, URL, PP, MC)

The word “Sex” itself is a taboo for us. (P5, F,45, MS, MPI, MML)

80% of the problems are cultural and caused by what society forces us to do against our human nature and preferences. It also has side effects, for example when a girl chooses to do things differently and has sex out of marriage, she will end up in my clinic for anxiety issues. “What if people know, what if I become pregnant, what if I get sick, what if my boyfriend won’t marry me and chooses a virgin”. See how lack of education and strong roots of social stigma and judgement ruin someone’s life? (P17, M, 43, PSY, PP, UM)

[Reason for not visiting a doctor for sexual health reasons] Embarrassment because they think others might think of them badly. This fear and shame of being judged stops them from visiting a doctor. (P1, F,60, OBGYN, UP, HPI, HML, MC)

Sexual health and sexual issues are yet to become normal in families here in Iran, the fact that you need to take care of your sexual health. (P9, F,47, OBGYN, PP, U)

2iii. Gender Inequalities

In fact, women are really passive in their sexual lives and they actually let their sex partners tell them what they want and these women would accept it no matter what. Maybe because they are afraid of getting rejected or losing their partners. Yesterday this young lady came to visit me, she told me that she wants to have sex and I told her that there is no problem but never forget to use condoms because it protects you from STIs but she told me that her partner doesn’t like to use it. I told her it doesn’t matter if he doesn’t like it, which one is the priority? Your health or his preference? But it was so obvious that she would choose her partners preference over her health. (P1, F,60, OBGYN, UP, HPI, HML, MC)

We live in a male dominant society so in most cases it's the male’s decisions that gets considered and men’s needs are the main priority and if the woman resists it might turn to violence against herself. So she has to give in. (P3, M,55, GP/MPH, HPI, HML)

Our women are used to say “yes sir” especially when they are in love or in a relationship, they give in easily. (P6, F,42, PhD)

When we ask women why don't you use condoms, they would tell that my partner doesn’t’ like to use it. (P15, F,30, MS)

In the case of women, they don't usually choose not using condoms themselves. They might try to teach their partners about some behaviors or get along with them and see what is comfortable for them however it doesn’t help much. Men feel they always know it all. (P17, M, 43, PSY, PP, UM)

2iv. Pre 2008 / 2013 Policy and Services

All services that sexual health centers provide used to be free. But after the recent policy aimed at increasing the population they don’t offer contraceptives and condoms for free anymore. Now the HIV trend is on the rise because they have stopped this service. (P1, F,60, OBGYN, UP, HPI, HML, MC)

We established an organization for registering and reporting STIs and we also planned and encouraged discussions about HIV nationally. In fact, I was the main policy maker we used to get consultation and help from various professionals who were active in academic sectors with different backgrounds and fields of study. (P3, M,55, GP/MPH, HPI, HML)

For STIs our main duty was controlling the diseases with focus on HIV. We have another department in Ministry of Health which is called Education Department. Health education to be more precise. They are the ones who make most policies for information and communication. In that period of time which I believe was the golden era, the reform years, HIV used to be a taboo and a stigma and you could’t talk about it comfortably, it was the same case for STIs. In this golden era the taboo was broken in a way that when I left my position after 10 years, they were discussing about teams that would go to different areas of the city and provide free HIV tests for young adults. We are talking the year 2006-7. Now it's all gone. (P3, M,55, GP/MPH, HPI, HML)

Talking about education, we came up with 5 minute teasers or 20 minute animations that had a specific funny character, it had 20 episodes with HIV prevention and safety tips as the main subject. It was broadcasted day and night on TV, the same TV that denies STIs exist today. Also we released 2 books, about what parents need to know about HIV prevention. I believe it was the first of its kind to make parents aware of such subjects. The book was published and was distributed only once, as we hit 2007 and it was removed from the market. I think it was a great combination with the TV teasers. Then we put forward the idea of having a step by step training in our schools with the Ministry of Education, we had many meetings and demanded all of these however it never got executed. We asked them to start by educating elementary school kids about their bodies and then expand the subject, even we offered to provide them with some headlines and topics however the Ministry of Education went through some policy changes at that time and all our efforts got shut down. We had come up with some effective topics and headlines that was approved by all of us and with a language that wouldn’t be offensive to anyone. (P3, M,55, GP/MPH, HPI, HML)

A council was formed immediately in the country. The president was the head of this council which was called “The High Council of AIDS”, however the successor of President Khatami decided to dissolve the council and kind of reversed all President Khatami did. So the council gave us the permission to build a clinic and pre-marriage classes were introduced. These 1 hour classes were about what contraception is or how long couples need to wait between pregnancies. STIs aren’t currently part of the pre-marriage tests. It used to be in the reform years. I’m not sure why but they stopped them. (P3, M,55, GP/MPH, HPI, HML)

We had places called “special consultation centers for vulnerable women”. In fact, we meant sex workers. Sex workers from more deprived areas or those who work in streets. We used to try to attract these people, we managed to bring 6000 to 7000 of them with 30 centers running in the whole country. Then we started a research on the conditions of sex workers in Tehran for the first time. It never got published and became classified. Then we did the same thing in Shiraz, the thing that we insisted on in "special consultation centers for vulnerable women” was that you should accept the fact that we do have sex workers because if you ignore this fact, these people would continue their activities underground then you won’t be able to get access to them. These people have to be accessible so that you can educate or treat them, we mentioned all of these in our instructions. We even created a booklet for sex workers without any logos and distributed it among them, there wasn’t anything similar to it so that we could just translate. We learned from sex workers and then provided for them. We held focus group discussions about what these people went through, these were the information that we wrote and executed, and all of these happened step by step. After leaving that position I have no idea how are things but everything kind of stopped and vanished after the Khatami era. I don't even know where the center for vulnerable women is. Hard liners were the ones who didn’t let it continue and they never will. (P3, M,55, GP/MPH, HPI, HML)

I can’t see why we are doing it to our own people. 10 years ago,15 years ago, same people received same care for free and with absolute respect and confidentiality. Now this is how it is. As if we go backwards instead of moving forwards. (P3, M,55, GP/MPH, HPI, HML)

In the past couples were encouraged to use permanent contraception methods such as vasectomy after having 2 kids however nowadays they just give a brief introduction for contraception methods but they don’t encourage it anymore. (P3, M,55, GP/MPH, HPI, HML)

I used to teach “Family planning” module at university. The one that I used to teach was about methods of family planning like pregnancy prevention with a more open perspective on the subjects. It also covered common STIs…. Right now if you go to any health center you can get contraceptives or condoms however under some conditions. Either you have to have a kid under the age of 2 or have 5 children or you should be 35 years old and have 3 children or you should be 40 years old and above or have a specific chronic disease. These conditions apply to all contraception methods including condoms. This is all after the policy to increase the population. Before that this all was free. (P6, F,42, PhD)

**3. Current Sexual Health Educational Provision**

Mainly in response to question 8 from the interview protocol.

8. What do you think about available sexual health education for young adults in Tehran, including pre-marriage classes and university modules?

From what I know in Iran there are specific centers under the supervision of ministry of health, they offer pre marriage classes. (P2, M,57, URL, PP, MC)

The main strategy of our national strategic plan is to educate and inform. We have target groups, each individual gets categorized into three groups of “high risk", “at risk” and “general population”. So we provide educational packages for each of these groups. These educational packages’ contents are simple and comprehensible, it starts with how HIV is an infectious disease and how it gets transmitted, how it should be treated and how it can be prevented. Anyone who refers to health centers and asks for it, can receive this education. (P5, F,45, MS, MPI, MML)

We have a center called “Youth center” that is under our control, we recruit high risk and at risk young adults there. It is developed by UNICEF’s “All in Project”. It is located in “Yaft Abad” which is a deprived area, when we started this “All In project” there were only 5 countries that had started this project and we were the first one across Middle East and EMRO area. Its public name is “Youth health center”. 50% of its fund comes from UNICEF and the other 50% is supplied from the national budget. This program teaches young adults about HIV. (P5,F,45, MS, MPI, MML)

We hold pre-marriage classes here. 2 hours of these classes are dedicated to sexual and pregnancy health. Around 90 minutes for ethics and religious rules, 45 minutes of legal rights and 90 minutes is dedicated to psychology. (P6, F,42, PhD)

There are 9 centers that are being covered by us (Iran University of Medical Sciences). 4 of these centers are placed in Tehran and 5 in other cities. There are other universities that hold such classes like “Tehran University” or “Shahid Beheshti University”. “Tehran University” has 3 centers in general but "Shahid Beheshti University” has more centers than us however I have no idea how many of these centers are placed in Tehran. They have 15 pre-marriage education centers. The classes are for free and are compulsory for everyone who wants to be married. They are single sex. (P6, F,42, PhD)

It is mostly around sexual organs and preparation for sex {in pre-marriage classes] unless they have other questions, at the end of class DICs (drop in clinics) and our AIDS centers are introduced so that if anyone is interested, they refer to one of these places to get themselves tested. (P6, F,42, PhD)

If they [young adults] refer to us by themselves, they will be advised [about HIV]. We also send our teams to schools in order to educate them particularly in occasions like world AIDS week. Other than these occasions, it is not compulsory for our teams to go to schools and teach kids. (P7, F,49, GP/ MPH, HPI, HML)

Based on protocols that’s been given to us there is a plan called “Health Ambassadors”. HIV is included in the contents that need to be taught in “Health Ambassadors” program so if it gets executed properly it would be great. We have to teach the trainers and trainers will transfer this information to the students and based on the goal that ministry of health team has set, trainers should choose and teach 10 percent of the students in each school to become health ambassadors themselves. We have provided different packages for different school levels in “Health Ambassadors” program, for example there’s a package specifically made for elementary students, one for middle school students and one for high school and our trainers should teach and educate students based on these packages. HIV has to be taught in Middle school and High school. In university level we also have “Health Ambassadors” scheme and HIV is more highlighted in this level, it also includes STIs other than HIV. (P7, F,49, GP/ MPH, HPI, HML)

**4. Limitations of Current Sexual Health Educational Provision**

Mainly in response to questions 8,9,11 and 12 from the interview protocol.

8. What do you think about available sexual health education for young adults in Tehran, including pre-marriage classes and university modules?

9. Do you think sexual health education in Tehran needs to be extended or changed?

a. If so, how?

11. Do you think sexually active young adults are in Tehran are protecting themselves against sexually transmitted infections?

a.What protection do you think they use?

12. How confident and in control do you think a typical 18-25 Tehranian 18-15 year old is in managing sexual relationships?

4i. Lack of Sexual Health Knowledge

As you might already know there’s no official education about this topic in schools and university, the only education that exist around this matter is pre-marriage classes which I have no clue who holds it and who the educators are. (P4, F,60, OBGYN, UAP, PP, UM)

There’s no official education around this topic in our schools. (P5, F,45, MS, MPI, MML)

So there’s no official education and it’s the people themselves who are after learning. (P5, F,45, MS, MPI, MML)

We don’t have any particular education in our schools and universities. (P6,F,42, PhD)

All I'm saying is if they know how to say no and how to manage their sexual life they have learnt it all by themselves. There has never been any motivation or education for that. (P8, F,55, OBGYN, UAP, PP, U)

Most young adults who come to my clinic don’t know even the most basic information…. So their information isn't reliable and basically one of the common questions that I get is what contraception method should they use, and even sometimes it happens that they ask how should they have sex or what position is better to go with. They also almost don’t know anything about STIs. (P1, F,60, OBGYN, UP, HPI, HML, MC)

Most of our referrals come here [center for behavioral diseases] for STIs testing, HIV specifically. Yet, they have no information whatsoever, regarding STIs or pregnancy or anything else related to their sexual and relationship health. (P16, F,55, MS)

There’s definitely a need for education so that young adult sexual health is improved or actually fixed. I can see recently that morning after pills are getting used more and more. There are women that have been married for years and they don’t know anything about it however I see that it is getting more common among our young adults. (P1, F,60, OBGYN, UP, HPI, HML, MC)

During the time that I was in contact with students, which was for the campaigns that we had for HIV awareness, I learned that everyone is eager to learn however they don’t know from where and how? Our problem is the lack of right sources of information. (P11, F,47, GP, HPI, HML)

I feel their knowledge is improving however it is not sufficient. For example, about their hormonal condition, sexual stuff, infections, transmission methods and STIs, their knowledge has improved a lot however you can see a lot of incomplete information among them. Like they don’t know all symptoms or all transmission methods but they know some. (P8, F,55, OBGYN, UAP, PP, U)

They are not educated about this stuff at all. For instance, a patient who wants to get married, imagine this person is a 26,27 year old. They come and say I want to have sex with my wife, where should I put my penis? Then I have to draw it to explain it to him. Because men have a literally external genitalia, they don't have much question about it but to explain the female body, I have to over explain everything. (P2, M,57, URL, PP, MC)

They need to get more educated but there aren’t any official resources or courses. (P9, F,47, OBGYN, PP, U)

4ii. Lack of Self and Relationship Management Skills

When I see young adults in our society I can see that they have heard about HIV and they know about the transmission methods but what is really interesting to me is that people have the knowledge but not the skill set to protect themselves. In fact, they only have information. (P5, F,45, MS, MPI, MML)

They don’t have the skills to use condoms. They don’t have the skills to say no. (P5, F,45, MS, MPI, MML)

With all this being said, with absolutely zero official education, our youth are doing great. They have tried and found information for themselves as much as they could. But then you can see it’s only information and no skills in cases like the current HIV transmission pattern shift. Unprotected sex is unfortunately the main way of HIV transmission currently. So even if there is some information, there is no behavior change behind it. (P5, F,45, MS, MPI, MML)

I think no one has the ability to manage their sexual relationships as they never been taught or encouraged to do so. (P3, M,55, GP/MPH, HPI, HML)

I believe our young adults don’t have the necessary skill sets to manage their sex lives at all because they haven’t been trained for it. (P5, F,45, MS, MPI, MML)

4iii. Pre-Marriage Provision

Education that is offered before marriage is 2 sessions that are basically a few hours, apparently they are extending the hours however I’m not so sure about it. They have come to realize further education is needed so they are increasing the hours, because unfortunately the divorce rate is really high in Iran and they have gathered sexual relationship dissatisfaction is the main reason. However, in these classes they don’t teach much so it's not sufficient [education]. (P1, F,60, OBGYN, UP, HPI, HML, MC)

I can say this without a doubt that 99% of them [those who have attended pre-marriage classes] have no idea that pre-ejaculatory fluid can lead to pregnancy. This should tell you how educated our young people are and how efficient pre-marriage classes are. (P16, F,55, MS)

Pre-marriage classes fail to work as they are redundant and useless. This is just sad really. (P10, F,48, MS)

These classes are better than nothing but are not really effective. They’re short and not much necessary content is covered in them. Contraception methods are currently removed from those classes due to recent policies. Also there is no evaluation of them. (P11, F,47, GP, HPI, HML)

I believe pre-marriage classes are useless and pretentious, they don’t teach anything practical and useful. And it’s not like they provide different methods for different people in accordance to their behavior, they have a specific model, a structured plan that they follow. In my opinion it hasn’t been effective. (P12, F,44, OBGYN, PP, MC)

I find the pre marriage classes absolutely absurd and useless. First problem is that these classes are in public so the person wouldn’t feel comfortable enough to ask their questions. The second problem is that it is mandatory so it means the person “has to” attend this class, another issue is the teachers, it doesn’t matter if the teacher has one class or 20 classes a day or even no classes they get the same salary. If these classes were operated by the private sector, the teachers had more income if they did their job better or got more clients and things would be different. It is also not the right time, couples are busy and under stress for their wedding but have to mandatorily come and sit this classes with no enthusiasm and because the age of marriage has increased these people already have some information from before, they may have received it online or from friends and god knows how much of it is wrong, they have lived their sexual life based on that information so the damage might be already done. So it is too late for these classes. (P3, M,55, GP/MPH, HPI, HML)

It might be effective for dumb people. The package is completely standardized and specified. You are not allowed to say anything out of the specified framework. I don’t have much information about pre-marriage medical tests, I just know that they are told about HIV and will be asked to get themselves tested if they want to, however we don’t have a full STIs test before marriage. The information provided [in pre-marriage classes] is so minimal that makes the whole thing so useless. (P5, F,45, MS, MPI, MML)

Some changes have been made to pre-marriage classes. Nowadays they have added subjects like rights, sharia laws and ethics, so in total it is 6 hours. But they have omitted contraception methods because of the increasing population policy. The classes were never effective, but now they are officially useless. (P7, F,49, GP/ MPH, HPI, HML)

The only thing that I’m sure about its existence is the compulsory pre-marriage classes which are for free, so every couple that decides to get married should pass these classes. Although I don’t think these classes are effective at all. (P8, F,55, OBGYN, UAP, PP, U)

The classes that are being held are only for a few hours. You can't get to the roots of a problem that is embedded in our culture in just a few hours. It may have some effects and could be a trigger and might push them to care more about their sexual health however it can’t change the culture. (P9, F,47, OBGYN, PP, U)

I never see a difference between my married and unmarried patients in terms of sexual health knowledge or safer sexual behavior. That can mean that these classes are probably nonsense. I haven't gone to these classes to check what they teach. Such classes should take place way earlier than that. Someone might want to get married at the age of 40. They will experience and do things without knowing what’s the right thing to do. (P13, M,42, INF, HPI, HML)

4iv. Organizational and Cultural Constraints on Improved SHRE

2 years ago we had a project called “prevention of risky behaviors in young adults”, in this project a questionnaire was provided by the ministry of health and we were supposed to give it out to 1200 students in north-west of Tehran because there are so many universities in that area. At first while giving the questionnaires to the students we told them that we don’t need any names or ID numbers, please just fill it in with honesty. Then an investigator from the ministry of health said that who knows, maybe you have faked all responses, you have to give us at least an ID number or a phone number. So except the first 200 questionnaires that were filled in with honesty and contained reliable data, the other 1000 questionnaires were filled in by people who never had a girlfriend or boyfriend or never used drugs or even never smoked hookah, which is obviously impossible. They were all so innocent that none of them had never watched porn on the internet, imagine that. I was like this project has already failed. You can’t get a reliable result when the students feel that there’s a little chance that they might get caught if they give out their identity. Because you can’t tell people you will contract HIV through unprotected sex and then interrogate them in surveys with names and ID numbers whether you have had unprotected sex. Things don’t work that way. (P10, F,48, MS)

High schools are restricted. Ministry of education doesn’t give permission for any action [regarding sexual health education]. Imagine kids in the first or second year of high school or even middle school, they are curious and full of questions and issues but there’s no health trainer or counselor to help them out. (P10, F,48, MS)

Some schools that are in affluent areas of the city might have counselors, however these counselors only encourage students to study more. Which means they only bring educational counselors and behavioral counseling is not their priority. So there is literally no education. They don’t care that kids have questions about their puberty, behaviors and emotions. They pretend that it doesn't exist. (P10, F,48, MS)

Right now we are planning to educate students about puberty in schools and they have already given us a few red lines that we should’t cross. (P10, F,48, MS)

You can’t ask someone not to do this or that, you have to tell them about the consequences of what they do. Right now if we have somehow managed to be successful against HIV is all because we talked about it, in the past we couldn’t talk about it openly and we could only mention it in some specific places within the ministry. Then they found out that it doesn’t work like that, you have to go out there and tell people the truth. (P10, F,48, MS)

We do have health ambassadors but unfortunately there’s no platform for them to do their job so to be honest it’s just show off. At first kids would be like “I will get a certificate” so they would be eager to participate but after going through all the trainings they would be like “why would I get myself into so much trouble”. I asked so many of these ambassadors that came here after their training about their activities but no one has given me a report yet. However, there might be some university students who are really interested but are too embarrassed or even scared to do anything at their universities because people would talk behind their back or label them. (P10, F,48, MS)

The concern of us collogues in the Ministry of Education was the parents of students not being prepared for such things, they would say you should start with the parents first and the explanation to their hypothesis was that imagine you teach something in a class let’s say condoms, when the student goes back home and tells their parents that today they had discussions and learned about condoms, because parents aren't ready, this would create conflict between parents and the schools. This is what concerned Ministry of Education the most at the time. They would say it’s not possible to start teaching kids before preparing parents. So the whole thing didn’t go forward. Even now in Tehran you can see that people’s priorities are diabetes or blood pressure instead of HPV. We have an almost young society however just look at the trends of our society, you see adverts related to blood sugar or blood pressure and no messages related to HPV, why? Because of the stigma that exists about these subjects, so you can’t talk about it openly and comfortably as you do about blood pressure. In schools if they decide to bring in a health speaker they would never ask them to talk about HPV, they want them to talk about nutrition or brushing your teeth. (P3, M,55, GP/MPH, HPI, HML)

Another issue is the stigma that is around HIV, even the person that teaches about it thinks of it as a taboo, it is true that this person has read the package contents but they are still not ready to teach it to others. Our almost nonexistent training programs are not effective because the teachers haven't been trained and our Ministry of Education is close minded, it is obvious that by using teachers who aren't updated and don’t have the necessary skill sets to deal with students, any program to educate young adults would be doomed to fail. Then young adults won’t learn to protect themselves. (P5, F,45, MS, MPI, MML)

Now we can’t really teach about pregnancy prevention because these [pre-marriage] classes are under surveillance and we have to talk and teach in line with the population increase policy. (P6, F,42, PhD)

It all depends on each health staff, some might be interested to go and teach in regular days and some don’t feel like to do it especially for private schools. I believe one of our main weaknesses is this, because health teachers’ salaries do not include HIV education so the teachers don’t feel motivated to go and teach and they prefer to spend their time in health centers instead. On the other hand, we don't have enough manpower to cover all schools so what happens is we have to prioritize. Some schools get the education and some won’t so we can’t claim that everyone is getting the HIV education. (P7, F,49, GP/ MPH, HPI, HML)

We don’t get enough budget in order to set up as many centers as possible with enough trainers and experts. (P7, F,49, GP/ MPH, HPI, HML)

Our education system doesn't let us go to schools to educate students about this stuff, the reason they give us is that the parents might complain about why did you tell this stuff to our naive children? They hadn’t heard about this before! (P15, F,30, MS)

Most of the decisions regarding this age group is being made by people who are from other generations and might not understand their issues in full capacity, so we need to communicate with them [young adults] to understand their real problems. (P10, F,48, MS)

What I am trying to say is that these people are not stupid. The problem is that we as legislators and policymakers who are in charge of education are underestimating these people. The content of what we teach is not even at the level of the comprehension of the rural kids, let alone others. We act so basic and we think we are doing a very good job. Education policy makers and execution of educational legislations and planning for students are not up to date at all. This is my conclusion from what I’ve seen during all this time that I’m working. This means legislators and educators are not up to date. (P11, F,47, GP, HPI, HML)

Officials and the society are 2 separate group in this matter which means there’s a huge gap between the people and the official organizations. (P5, F,45, MS, MPI, MML)

4vi. Lack of Formal Evaluation of Programs and Content

No official evaluation has been done to see how effective these classes [pre-marriage] are. So it’s a broken cycle being constantly repeated. (P3, M,55, GP/MPH, HPI, HML)

There’s also no evaluation or appraisal. They’ve only kept doing it [pre-marriage classes] for the past 10 years or so. (P5, F,45, MS, MPI, MML)

Our monitoring covers different areas. A part is checking the teaching methods and a part is checking the quality of venue and educational tools used and finally checking the quality of information with a series of questions. After these classes we don’t have any access to the couples to observe the effectiveness of these classes so our only way is to see if they were satisfied with the classes or not right after it. We ask a few questions to see if they think the education that they got was good and sufficient however to determine the real outcome of these classes we haven’t had any access to any of them afterwards and unfortunately no research has been done about it and all we've done was "post testing” at best. This can’t be a real evaluation. (P6, F,42, PhD)

We just educate them [in pre-marriage classes] but evaluation is not something we have ever done. (P7, F,49, GP/ MPH, HPI, HML)

We have only opened centers for behavioral diseases but we have never assessed how many people know of these centers or refer to them on a monthly basis. (P11, F,47, GP, HPI, HML)

Also there is no evaluation of them [pre-marriage classes]. It would be awesome if someone would come and do an evaluation to see what issues these couples have faced after a few years of passing these classes? And how they have managed to solve them? (P11, F,47, GP, HPI, HML)

**5.** **Informal Sexual Health Education and Their Limitations**

Mainly in response to questions 3 and 8 from the interview protocol.

3. How well educated do you think young adults in Tehran are in relation to sexual health (e.g., in relation to prevention of unwanted pregnancy and sexually transmitted infections)?

8. What do you think about available sexual health education for young adults in Tehran, including pre-marriage classes and university modules?

5i. Friends

Young adults know some stuff but not much, and they have learned it from their friends. They only use withdrawal. They only do it because their friends do it. (P2, M,57, URL, PP, MC)

When there’s a problem people usually first look for a solution from the people they know and if it doesn’t get resolved they refer to us as a last resort. They first ask their friends. (P17, M, 43, PSY, PP, UM)

They find out about us [center for behavioral diseases] from friends or peer groups. (P14, M,35, BA)

They have heard and learned about contraception methods from others and those others have heard it from other people as well. (P1, F,60, OBGYN, UP, HPI, HML, MC)

They talk to their friends about it [ their sexual health issues]. (P2, M,57, URL, PP, MC)

They receive education and information from other sources. Like their friends. (P5, F,45, MS, MPI, MML)

Most of the information they get is from friends and word of mouth. Actually our young adults are discovering things on their own. They're not so well educated. (P2, M,57, URL, PP, MC)

5ii. Internet and social media

Kids who are embarrassed to ask their questions from grown-ups would reach out to social media to get their answers. (P10, F,48, MS)

There is an increasing use of internet and social media and it has contributed to people’s sexual knowledge to an extent however it doesn't mean their knowledge is correct. (P13, M,42, INF, HPI, HML)

Nowadays our young generation rely on social media. (P3, M,55, GP/MPH, HPI, HML)

Their main source of information is definitely Internet. (P4, F,60, OBGYN, UAP, PP, UM)

Internet. Like social media and online forums. (P5, F,45, MS, MPI, MML)

All the information is on social media. They will learn where they can find the most reliable information. They will learn it like all the other things they do. The society is helping itself with no official support. (P5, F,45, MS, MPI, MML)

The general population is getting itself educated by using social media. (P5, F,45, MS, MPI, MML)

Nowadays almost everyone has access to some kind of information thanks to the Internet however you can’t distinguish right information from wrong information. I think our young adults get their needed information from the Internet and therefor reliability of the information they receive is an issue. (P7, F,49, GP/ MPH, HPI, HML)

Almost all my patients google their questions. (P13, M,42, INF, HPI, HML)

We live in the age of WhatsApp and Instagram. If you as a mother don’t inform your kid, she/he would go and get all sorts of wrong and unreliable information on Instagram. (P15, F,30, MS)

People turn to Instagram and Telegram to learn absolutely unreliable stuff. (P17, M, 43, PSY, PP, UM)

No one knows that many of these information is wrong and the interesting part is that the websites that provide false information are more attractive and know how to get the attention of users by focusing on the action of sex not sexual health. Especially with most contents being blocked. Blocked contents create more curiosity, the youth want to know what is this secret that is blocked. (P10, F,48, MS)

They mostly use online resources and since not all the online data can be reliable, this has become troublesome. (P12, F,44, OBGYN, PP, MC)

Social media has its pros and cons. You are exposed to right information and the wrong ones and you can't ask questions. You see something and some questions will pop up into your mind. It is one sided and you can't distinguish the right information from wrong ones. (P3, M,55, GP/MPH, HPI, HML)

But then the internet is like an ocean, anything could be found in it and many sources of information could be unreliable or misleading. (P4, F,60, OBGYN, UAP, PP, UM)

God knows how reliable is the information that I would find by myself, either online or elsewhere. (P5, F,45, MS, MPI, MML)

Considering social media, I think the amount of false information that they get is much more than the right ones. (P6, F,42, PhD)

Right now social media is contributing to misconceptions. If only they could put some sort of control over these [Telegram]channels we wouldn’t have this much of false information among our youth. (P6, F,42, PhD)

How much of this information [online resources] is reliable? How much of it is true and how much of it helps their decision making about their health and relationships? It all depends on luck. (P16, F,55, MS)

Also Internet has had both positive and negative effects. The positive effect is that they have access to educational videos on YouTube. People can learn things from it however the negative effect of the internet is the incomplete knowledge of STIs and the fear that comes with it. There is so much unreliable information on STIs and we have patients coming to us in fear because they think that random rash is now a HPV symptom. And they won’t believe us when we assure them it’s a simple rash or allergic reaction. They keep saying but google told me this is a serious illness. (P2, M,57, URL, PP, MC)

**6. Sexual Health Services for Young People**

Mainly in response to questions 5 and 10 from the interview protocol.

5. How adequate/comprehensive do you think sexual health services are for young adults in Tehran?

10. What do you think about available sexual health services for young adults in Tehran?

a. Do you think these are known of and accessible to most young adults in Tehran?

6i. Services in Center for Behavioral Diseases

Other than counseling, free HIV test is provided to them however other STI tests are not included. Here I give MSMs couple therapy or individual therapy. (P14, M,35, BA)

Here we give them free condoms. Our services are free for everyone, but all we ever see is super high risk population, like low end male or female sex workers. High end female sex workers go to our other center, which is designed for them only. (P14, M,35, BA)

There is not much use in them [center for behavioral diseases] for the general population, mostly high risk people attend them. (P11, F,47, GP, HPI, HML)

When people come here to get tested, they first go through a counseling session and then we test them for HIV based on the risky behaviors they’ve told us about during that process. We take HIV rapid tests however our main focus is on counseling. Our counseling process works like this, the person refers here and tells us that he/she has had unprotected sex. Either with a sex worker or a partner. Based on this information, we can tell if getting tested is necessary or not. If they used condoms every time they had sex then these people don’t need to get tested however if they had unprotected sex even once, we will take the test. So it goes like this: have you ever had unprotected sex? If yes, you need to take the rapid test. Or sometimes the person comes here and tells us that I shared a needle with someone else, whether it was for using drugs or injecting hormones in the gym, these people are considered as high-risk, it goes the same with children with HIV positive parents. If the result of the rapid test comes out positive they are sent for further clinical tests. We only do HIV tests here and other STIs are not screened in this center. People will need to go to a specialist if they have other STIs symptoms. (P15, F,30, MS)

The center for behavioral diseases doesn’t do much [for general population], because it has become kind of exclusive to high risk people. (P10, F,48, MS)

6ii. Funding Limitations

Health budget is mostly focused on high risk groups. For the general population there are not much done in terms of sexual health. (P7, F,49, GP/ MPH, HPI, HML)

Unfortunately, at the moment all of our sexual health services, which are limited compared to the other countries, are only tailored for the high-risk groups. (P14,M,35,BA)

If a person is a sex worker our health centers would provide them with free condoms, however the general population should pay for it and there are some people who might not have anything to eat then imagine they should pay 30000 Tomans (around 2 pounds) for a package of condoms that they don’t know if it is of good quality or not, so they would ignore it. Therefore, it is expensive for everyone unless you are a sex worker. We should beg our sex workers to take free condoms and use them. But for regular people, they come and beg us for condoms and we can’t give them anything, as we are told not to. (P6, F,42, PhD)

**7. Barriers to Seeking Sexual Healthcare**

Mainly in response to questions 5,10,13 and 14 from the interview protocol.

5. How adequate/comprehensive do you think sexual health services are for young adults in Tehran?

10. What do you think about available sexual health services for young adults in Tehran?

a. Do you think these are known of and accessible to most young adults in Tehran?

13. Can you identify barriers to young adults seeking sexual health knowledge and/or sexual healthcare in Tehran?

14. Do you think sexual health care (including contraception pills, condoms, educational materials, visits to doctors etc.) for young adults in Tehran is inexpensive or expensive?

7i. Lack of Publicity for Government Funded Sexual Healthcare Facilities

Maybe 10% know of such places. And those are the ones who’ve studied health sciences or medicine as they kind of are in related fields. There is a budget dedicated to the “Center for Behavioral Diseases” but no one knows where it is and therefore it’s become a center for high risk people. I think mostly sex workers would refer to this centers. (P6, F,42, PhD)

There are health centers but the biggest problem is that unfortunately almost no one is aware that these facilities exist. In other words, there’s no publicity and promotion for these facilities. (P1, F,60, OBGYN, UP, HPI, HML, MC)

Regarding sexual healthcare facilities, there might be some centers but I don’t know about them. (P12, F,44, OBGYN, PP, MC)

They say these centers exist however even I as a doctor don’t know where they are, let alone the regular citizens. (P4, F,60, OBGYN, UAP, PP, UM)

They are not publicized, as I said earlier, if someone wants to find something they will, otherwise there is no direct advertisement for such centers. (P5, F,45, MS, MPI, MML)

Your workshop could be a starting point to make such centers public, as there's never been any advertisement for them. (P7, F,49, GP/ MPH, HPI, HML)

I don’t know maybe there are places however they don’t really encourage people to refer to them. There is no advertisement or anything. (P8, F,55, OBGYN, UAP, PP, U)

Unfortunately, the general population doesn’t know about us [ the center for behavioral diseases], so we don’t see much regular people here. (P14, M,35, BA)

So many people don’t know about our center [center for behavioral diseases]. (P15, F,30, MS)

7ii. Costs

Unfortunately, the truth is visiting a doctor is expensive. I believe sexual health and education should be free. I believe all contraception methods should be offered for free to everyone, just like before. (P1, F,60, OBGYN, UP, HPI, HML, MC)

It [sexual healthcare] must be offered for free. Now the prices are really high and it is expensive. (P2, M,57, URL, PP, MC)

I believe it all depends on the socioeconomic levels of each individual, however I think people are now more willing to spend to protect themselves compared to ten years ago. And I believe the cost is more justified to them than before. But still so many people might not be able to afford it. We have no statistics of how many people buy contraceptives regardless of costs. (P3, M,55, GP/MPH, HPI, HML)

To be honest contraceptives and condoms are cheap in comparison to other stuff, but do people prioritize it to food and other commodities? I don't think so! (P4, F,60, OBGYN, UAP, PP, UM)

Yes, it is costly. Not so many can afford it. For example, a young girl who does’t have a job and gets pocket money from her parents should pay around 250000 Tomans (around 15 pounds) for these tests. HIV tests are really expensive. In general, the tests that we ask our patients to have such as VDRL, HIV or Hepatitis C are very expensive. (P4, F,60, OBGYN, UAP, PP, UM)

The cost [for sexual healthcare and contraceptives] is unreasonably expensive. (P8, F,55, OBGYN, UAP, PP, U)

They are [sexual healthcare and contraceptives] relatively expensive, especially in the current economic situation. (P9, F,47, OBGYN, PP, U)

Those who can afford it, refer to private clinics and doctors, and receive all care needed, at times even illegally, like abortion. Those who can’t, the absolute majority, suffer in silence. (P3, M,55, GP/MPH, HPI, HML)

Expenses are the main barrier to seeking sexual health care and contraception. Not everyone is affluent and people would rather prioritize their other needs to sexual healthcare or condoms. (P7, F,49, GP/MPH, HPI, HML)

Healthcare in general is really expensive nowadays and since there are no government funded sexual health clinics, or maybe there are and no one knows about them, people have to see private doctors and that is really expensive. (P8, F,55, OBGYN, UAP, PP, U)

7iii. Inequalities in Sexual Health and Care Seeking

Now that this taboo [sexual matters] is broken [ for affluent people], people’s knowledge has increased. Although as I said earlier, it is still a taboo for poor people. For those who struggle financially it still is a taboo. This is kind of a gap of its own, because the socioeconomics have grown people so apart that even in their cultural and personal beliefs the affluent are so progressive and different. (P4, F,60, OBGYN, UAP, PP, UM)

One reason [for not visiting doctors] is embarrassment for lower class citizens as sex amongst them is still a taboo. And then it is cost of sexual healthcare, again for the same social class. I don't think affluent citizens face any barriers. (P4, F,60, OBGYN, UAP, PP, UM)

It all depends on their social class and their education level [ whether they’re sexually educated or not]. People who are well educated and are from upper classes have a better condition. However, from what I see people in deprived areas don’t have much information compared to the affluent ones. Therefore, I believe people who live in more deprived areas of Tehran have less knowledge. It is right that they have access to the internet and cellphones but maybe they don’t refer to the right sources. (P8, F,55, OBGYN, UAP, PP, U)

We are in an affluent area of Tehran so normally I would expect people who refer to us to have a better level of knowledge in sexual health. They do have more knowledge in comparison to those who live in deprived areas. (P9, F,47, OBGYN, PP, U)

7iv. Distrust in Available Services

And those who want to learn don’t trust us. We are not trustworthy in their eyes. Unfortunately, our health system hasn’t managed to introduce itself to all levels of society with a good impression. Now look at the whole country and you wouldn’t find even one high school student that would refer to us to ask their sexual questions. They wouldn’t. Because we still haven’t managed to gain their trust and make them aware of our full range of services. There no trust, That’s why if you are a university or high school student and have an issue or even want to have a sexual relationship you wouldn’t refer to a doctor like me and say: I want to have sex with my partner, what should I do? You would Google it. (P11, F,47, GP, HPI, HML)

Also they don’t trust these services [center for behavioral diseases], they don’t trust them being confidential and non-judgmental. It means people find internet and their friends trusted sources instead of us. That’s sad, but once we have lost this trust, we can’t gain it back so easily. We never had that trust in the first place, so I really don't blame people for not trusting us. (P11, F,47, GP, HPI, HML)

We can’t communicate or gain the trust of our young adults who are at risk of getting STIs as long as we carry an official name and logo with us because officials have failed to gain society’s trust. Because we don't approve and accept different groups that live within this society. …. I even once mentioned it in a national committee meeting that students can’t trust their teachers. Because they are scared that they would tell their parents or even other teachers. Our young adults are interested in getting educated, they have a lot of questions. However, they don’t find someone who they can fully trust to ask these questions from. (P5, F,45, MS, MPI, MML)

**8.** **Recommendations for Improved Sexual Health Education and Services in Tehran**

Mainly in response to questions 9 and 15 from the interview protocol.

9. Do you think sexual health education in Tehran needs to be extended or changed?

a. If so, how?

15. What are your top five recommendations for improved sexual health education and training in Tehran?

8i. Creation of Official Sources of Information

Our general population is getting wrong information from unreliable sources online, however, we can change that by guiding them to use reliable sources. We can provide reliable information sources online, so that we can encourage them to go to these websites and learn about things anonymously and privately. (P14, M,35, BA)

I believe the ministry of health or education should start a serious educational or awareness raising campaign and they have to provide websites or (Telegram) channels to educate people about sexual health. (P1, F,60, OBGYN, UP, HPI, HML, MC)

This is where the policy makers need to step in, they don’t want face to face communication about sex? Fine, tell our children what online source is reliable and educative and they will find the way. (P4, F,60, OBGYN, UAP, PP, UM)

I really believe in social media and the internet. I would have developed reliable sources on social media and internet. I would have introduced reliable online sources to young people and would have asked them to go learn things there confidentially and comfortably. (P5, F,45, MS, MPI, MML)

Young adults constantly search and find a variety sources of information. We need to at least tell them about reliable sources. (P11, F,47, GP, HPI, HML)

8ii. Official School / University Based SHRE

I believe this education needs to be started gradually from the end of elementary school and the beginning of high school years in all classes. Pre-marriage is just too late. (P1, F,60, OBGYN, UP, HPI, HML, MC)

If I was in charge of this country’s education planning, I would choose pre-school and elementary school to start teaching about sexual health. (P17, M, 43, PSY, PP, UM)

If I was in charge of policy making and education planning, I would start a sexual health course in schools. I would start it from age 10. When it becomes part of the curricula, exams will be taken and level of knowledge would be examined so if there's any gap in the education or communication method, it can be worked on. I would have included a chapter on consent and importance of sexual choices being a private issue that no one can judge or critique. (P1, F,60, OBGYN, UP, HPI, HML, MC)

We need to find a solution so that we can start the [sexual health] education from high school level. (P10, F,48, MS)

In my opinion it all should start in school. If they learn this information they can be saved from so many diseases such as HPV and herpes. (P2, M,57, URL, PP, MC)

It should start from high school. I believe kids need to be educated from the age of 12 or 13. For sexual organs and anatomy I would start then and later in high school I would move to sexual hygiene and sexual health and self-care. (P4, F,60, OBGYN, UAP, PP, UM)

We need serious and rigorous sexual health education right after elementary school. We need to teach them a comprehensive course and let them ask all their questions. But I’m not the one who sets policies, unfortunately. (P5, F,45, MS, MPI, MML)

Sexual health education should start at elementary school and before they hit puberty. I believe sexual health is a priority. (P9, F,47, OBGYN, PP, U)

If I were a legislator, I would start it from the elementary school. SHRE should be given to elementary school students so that they learn things from a young age and learn to live with it. I think our educational system is way out of date. Teachers haven’t been trained about these stuff so children can’t talk to them or ask them even about puberty. (P6, F,42, PhD)

It would be effective if it was an early and continuous education because a single session is not effective enough to change one’s behavior and form of thinking, if we want it to be effective it should be continuous and ongoing. We are in need of public health and sexual health specialists to take over this role. (P6, F,42, PhD)

It should start from elementary school. Considering the increasing number of sexual abuse and child molestation cases, I think we have to teach our children about their personal privacy and how much they should let a stranger get close to them, I would start teaching these stuff very early. Then I would teach them about how they can protect themselves, differences between male and female bodies so that they don't think of the opposite sex as something scary and alien. I believe elementary school is the best time to start educating kids however it should be in accordance to their age. I think it should get started in elementary school and then be continued until university. (P8, F,55, OBGYN, UAP, PP, U)

**9.** **Support for a New Workshop**

Mainly in response to question 16 from the interview protocol.

16. Do you think a drop-in morning or afternoon workshop for young adults in Tehran on protection against sexually transmitted infections and unwanted pregnancy would be a good idea?

a. If such a workshop were to be run what would you recommend as the top five content areas it should cover?

Everyone’s been waiting for such a thing to happen. They will show interest. (P3, M,55, GP/MPH, HPI, HML)

They’re craving such a thing. If anyone knows there is such a workshop going on, they will come running. (P5, F,45, MS, MPI, MML)

Honestly I think everyone would love to attend such a workshop. There are no alternatives. This is like a fresh air, a new idea. Something they have really wanted for so long. (P6, F,42, PhD)

Everyone would love free extra information. I see no barrier to it whatsoever. They will learn about sexual health and they will receive information for sexual health centers. It's free and its educative, this can’t get better. (P7, F,49, GP/ MPH, HPI, HML)

I think it’s a great idea. (P1, F,60, OBGYN, UP, HPI, HML, MC)

That would be cool. (P11, F,47, GP, HPI, HML)

Oh yes, anything is good as there is no other alternative. (P4, F,60, OBGYN, UAP, PP, UM)

Honestly I think everyone would love to attend such a workshop. There are no alternatives. This is like a fresh air, a new idea. Something they have really wanted for so long. (P6, F,42, PhD)

It would be great. (P7, F,49, GP/ MPH, HPI, HML)

Yes, although 2 hours is not enough, it could be a good start. (P13, M,42, INF, HPI, HML)

Yes, it sure is [helpful]. Any reliable education in our country’s current situation, where there is absolutely no education, is good in my opinion. (P14, M,35, BA)

Based on what we see here, yes, definitely would be helpful. (P15, F,30, MS)

**10.** **Content Suggestions for a New Workshop**

Mainly in response to question 16 from the interview protocol.

16. Do you think a drop-in morning or afternoon workshop for young adults in Tehran on protection against sexually transmitted infections and unwanted pregnancy would be a good idea?

a. If such a workshop were to be run what would you recommend as the top five content areas it should cover?

10i. Anatomy of Sexual Organs

The first thing that definitely needs to be taught is the anatomy of sexual organs. I would explain it in a way that is simple and easy to understand. They should learn about what happens during sex and how does pregnancy happen. (P1, F,60, OBGYN, UP, HPI, HML, MC)

One of the things that I would teach about in the workshop would be sexual organs. (P15, F,30, MS)

First should be sexual organs. (P2, M,57, URL, PP, MC)

Sexual organs and anatomy. (P3, M,55, GP/MPH, HPI, HML)

[Sexual] Anatomy. (P5,F,45, MS, MPI, MML)

Sexual organs and anatomy. Puberty and body changes, periods, importance of having them regularly and importance of missing periods and therefore visiting a doctor for It. (P9, F,47, OBGYN, PP, U)

They should learn and get familiar with their sexual organs. (P17, M, 43, PSY, PP, UM)

10ii. Pregnancy Prevention, STIs Protection and Condom Use

How conception happens, how to aid or avoid it, how to keep healthy against STIs, and should that happen, who to go to and how to fix things. What STIs are out there and how are each transmitted. What treatment options or protection methods are available and why is it so important to buy, carry and use condoms. (P16, F,55, MS)

I would teach them about sexual health, STIs symptoms and transition methods. (P15, F,30, MS)

STIs are issues related to sexual relationships therefore they need to learn about them. (P1, F,60, OBGYN, UP, HPI, HML, MC)

I would teach them about protection against STIs and contraception methods, and also about different STIs and their transmission methods. (P14, M,35, BA)

So the content would be STIs symptoms and prevention and pregnancy prevention (P10, F,48, MS)

STIs and contraception. Because these are the real basic and important ones if you only have 2-3 hours. (P6, F,42, PhD)

I would definitely teach them about STIs. I would teach them about having a healthy and protected sexual relationship. I would teach them about the protection methods, then I would talk about their concerns and problems that they might face and I would explain what they should do next if something happens while they were under the influence of some drugs or have unprotected sex. If they were at risk, what should they do to reduce the damage or stop it from escalating. Couples need to learn about contraception methods together. I think having a general and comprehensive knowledge about contraception methods would be helpful. Then they can choose what method is best for them to choose. (P11, F,47, GP, HPI, HML)

Then I will cover contraception methods and how each have cons and pros. Then STIs and their symptoms. Then I would add that they need to get vaccinated for HPV and get tested for STIs. (P12, F,44, OBGYN, PP, MC)

They should learn about STIs. (P2, M,57, URL, PP, MC)

STIs prevention and protection and then pregnancy and contraception. We constantly hear young adults saying” Oh I never knew you can get pregnant that way” or “Wow, so HIV could be transmitted that way too? “So yea, this is the main topic. (P5, F,45, MS, MPI, MML)

STIs prevention and symptoms. Contraception of all kinds and how serious is the risk of STI transmission and unwanted pregnancy. (P3, M,55, GP/MPH, HPI, HML)

Pregnancy prevention because there is no other class teaching that. STIs, their symptoms and methods of transmission need to be discussed. STIs are important. Making sure that if they are going to have sex, they use condoms and make sure their partners are healthy through regular tests. (P7, F,49, GP/ MPH, HPI, HML)

Contraception methods so if they happen to have sex they don’t end up going through unwanted pregnancy, abortion and things like that. The other important subject to teach them is STIs which is really common nowadays. How can they avoid them and protect themselves against them, whether with vaccination or using condoms? Introducing places that they can refer to in order to get vaccinated or tested for STIs in Iran and encourage them to always use condoms in order to prevent the transmission of STIs. (P8, F,55, OBGYN, UAP, PP, U)

If I had 2 hours, I would teach them about protecting themselves, in fact I would teach them how to have a safe sexual relationship. I would tell them to buy and use condoms. Some people have the money to buy condoms but they prioritize other stuff that are not necessary. We should teach this stuff however you can't do it all in just 2 hours. Condoms will be my main suggestion. So my advice would be using condoms every time at all cost and purchasing it even if they have to sacrifice mini pleasures. Then I would teach them about STIS’ transmission methods. (P13, M,42, INF, HPI, HML)

They should be taught about using condoms, then they should learn about STIs and their symptoms and when they need to see a doctor for it and also pregnancy prevention. (P4, F,60, OBGYN, UAP, PP, UM)

First thing should be unprotected sex which is really important to talk about and learn to avoid. STIs and their symptoms and transmission methods and how common they are. What are the risk factors and how could they be avoided or limited? Contraceptive methods and their failure rates, which methods are out there and who should choose what method. (P9, F,47, OBGYN, PP, U)

Also teach them about all STIs, prevention and protection methods, treatment options and also contraceptive methods and consistent condom use. (P17, M, 43, PSY, PP, UM)

10iii. Provision of Contact Details for Available Sexual Healthcare

I would also include addresses and contact details of our centers in that workshop, in case anyone wants to come over. (P15, F,30, MS)

I would also provide them with this center's contact details so that they could visit us if they ever need any sexual healthcare. (P16, F,55, MS)

I would tell them the contact details and locations of sexual health centers in case they need to refer to a specialist. (P13, M,42, INF, HPI, HML)

10iv. Self and Relationship Management Skills

I would at least educate them about how a healthy sexual relationship looks like, how it can happen and what are the basics for it. Why should we learn to say no to persuasive and pushy people? Why should we always use condoms, carry condoms and don’t expect it from the other person. Care for yourself. Things like this. They still have to learn to say no. It's ok not to want to have sex with someone only to keep or please them, our schooling system creates robot like humans who constantly say yes sir, and can’t disagree or say no without feeling there will be consequences. You should give them advice on how worthy and important their health is and that they shouldn’t gamble it on keeping a relationship going. Neither they should give in because of embarrassment or their ego. (P12, F,44, OBGYN, PP, MC)

I believe now the most important subject that needs to be covered is the management of sexual relationships which is really important for both sides. (P3, M,55, GP/MPH, HPI, HML)

Consent and healthy decision making. (P6, F,42, PhD)

Self-protection and learning to say no, so that they won’t get STIs. (P7, F,49, GP/ MPH, HPI, HML)

Social and communication skills, like learning to have the courage to say no even if there is a risk that they would lose the person’s interest or the relationship all together. (P8, F,55, OBGYN, UAP, PP, U)

We should put our focus and attention on topics like emotional regulation and creating health motivation for individuals. STIs and health education is the first step for sure, however teaching emotional regulation methods should follow after. They need to learn that they should’t sacrifice their life and future for an instant pleasure by taking high-risk actions. Other than these they need to learn about negotiation skills and the skill to say no. This is way beyond the scope of your intended workshop, but if you ever wish to continue those workshops to more than a single session, I believe what I said is a must. (P14, M,35, BA)

**11. Workshop Delivery Suggestions**

Mainly in response to question 16 from the interview protocol.

16. Do you think a drop-in morning or afternoon workshop for young adults in Tehran on protection against sexually transmitted infections and unwanted pregnancy would be a good idea?

a. If such a workshop were to be run what would you recommend as the top five content areas it should cover?

11i. Mixed or Single Gender Classes

First is gender separation when it comes to such stuff because these are sensitive subjects in our culture and not everyone is comfortable with them. You will definitely need to ask your audience whether they want to be in mixed or single gender settings for such education. (P10, F,48, MS)

Well, usually religious women don’t like to participate in mixed gendered classes. (P8, F,55, OBGYN, UAP, PP, U)

I believe in order to make them attend comfortably and ask their questions without hesitation these classes would be better to be separated for each gender. (P8, F,55, OBGYN, UAP, PP, U)

These classes should never be single-gender. (P16, F,55, MS)

Mixed gender classes might make them feel uncomfortable and it may stop them from attending the class. Although if they've specifically asked for mixed-gender classes, then they would show up. (P13, M,42, INF, HPI, HML)

I suggest that classes shouldn’t be mixed-gendered, at least at basic levels because it will become a speed-dating situation, I mean it will surpass education as the main purpose, so I don’t suggest it. (P17, M, 43, PSY, PP, UM)

The workshop should be mixed gender. (P4,F,60,OBGYN,UAP,PP,UM)

11b. Group Discussions and Q&A

Engage them and let them interact with you and with one another. (P10, F,48, MS)

Ask them to tell you what else they want to hear. Or maybe they want to ask questions for the rest of 1.5 hours. Let them do that. A lot could be understood from their questions. Maybe their priority is different to what we deem as priority. (P3, M,55, GP/MPH, HPI, HML)

… And then a certain amount of time for questions and answers. Let them ask even the stupidest questions they may have. Let them feel it's ok to ask and not be judged for it. (P5, F,45, MS, MPI, MML)

You should allow time for discussion and questions. This way they will learn from each other as well. (P4, F,60, OBGYN, UAP, PP, UM)

Then let them ask any questions they might have and let them be as open as they wish to be. It's their first chance in their lifetime. (P17, M, 43, PSY, PP, UM)
